# gC1qR/HABP1/p32 Is a Potential New Therapeutic Target Against Mesothelioma

**DOI:** 10.3389/fonc.2020.01413

**Published:** 2020-08-12

**Authors:** Ellinor Peerschke, Kenneth Stier, Xiaoyu Li, Evelyn Kandov, Elisa de Stanchina, Qing Chang, Yuquan Xiong, Katia Manova-Todorova, Ning Fan, Afsar Barlas, Berhane Ghebrehiwet, Prasad S. Adusumilli

**Affiliations:** ^1^Department of Laboratory Medicine, Memorial Sloan Kettering Cancer Center, New York, NY, United States; ^2^Departments of Medicine and Pathology, Stony Brook University, Stony Brook, New York, NY, United States; ^3^Department of Thoracic Oncology, West China Hospital, Sichuan University, Chengdu, China; ^4^Department of Surgery, Thoracic Service, Memorial Sloan Kettering Cancer Center, New York, NY, United States; ^5^Memorial Sloan Kettering Cancer Center, Sloan Kettering Institute, New York, NY, United States; ^6^Molecular Cytology Core Facility, Sloan Kettering Institute, Memorial Sloan Kettering Cancer Center, New York, NY, United States

**Keywords:** mesothelioma, complement, gC1qR/HABP1/p32, monoclonal antibody therapy, therapeutic target

## Abstract

Mesothelioma is an aggressive cancer of the serous membranes with poor prognosis despite combination therapy consisting of surgery, radiotherapy, and platinum-based chemotherapy. Targeted therapies, including immunotherapies, have reported limited success, suggesting the need for additional therapeutic targets. This study investigates a potential new therapeutic target, gC1qR/HABP1/p32 (gC1qR), which is overexpressed in all morphologic subtypes of mesothelioma. gC1qR is a complement receptor that is associated with several cellular functions, including cell proliferation and angiogenesis. *In vitro* and *in vivo* experiments were conducted to test the hypothesis that targeting gC1qR with a specific gC1qR monoclonal antibody 60.11 reduces mesothelioma tumor growth, using the biphasic mesothelioma cell line MSTO-211H (MSTO). *In vitro* studies demonstrate cell surface and extracellular gC1qR expression by MSTO cells, and a modest 25.3 ± 1.8% (*n* = 4) reduction in cell proliferation by the gC1qR blocking 60.11 antibody. This inhibition was specific for targeting the C1q binding domain of gC1qR at aa 76–93, as a separate monoclonal antibody 74.5.2, directed against amino acids 204–218, had no discernable effect. *In vivo* studies, using a murine orthotopic xenotransplant model, demonstrated an even greater reduction in MSTO tumor growth (50% inhibition) in mice treated with the 60.11 antibody compared to controls. Immunohistochemical studies of resected tumors revealed increased cellular apoptosis by caspase 3 and TUNEL staining, in 60.11 treated tumors compared to controls, as well as impaired angiogenesis by decreased CD31 staining. Taken together, these data identify gC1qR as a potential new therapeutic target against mesothelioma with both antiproliferative and antiangiogenic properties.

## Introduction

Mesothelioma is an aggressive cancer of the serous membranes, typically those lining the pleural space (1, 2). It is chiefly caused by exposure to and inhalation of asbestos. Treatment outcomes continue to be poor, despite multimodal therapy consisting of surgery, chemotherapy, and radiation (3–5). An estimated 38,400 individuals die globally each year from mesothelioma (6), and the incidence is expected to rise in the US as a result of asbestos exposure following destruction of the World Trade Center in New York, NY in 2001 (7). Novel therapies, especially targeted therapies, are needed to improve treatment outcomes and reduce off-target side effects (8, 9).

The complement system is emerging as a novel target in cancer therapy. Complement is involved not only in shaping the inflammatory tumor microenvironment, but also in tumor growth and spread (10). In this regard, the complement component C1q is increasingly recognized as a tumor-promoting factor. It has been reported to enhance cancer cell adhesion, migration, proliferation, and angiogenesis (11–13).

We have identified gC1qR (also known as p32/HABP1) as the major cellular binding site for C1q (14). Marked upregulation of gC1qR expression has been observed in cancers of epithelial cell origin including breast, colon, and lung cancers (15, 16). In patients with breast cancer (17, 18), prostate cancer (19), and serous ovarian adenocarcinoma (20), as well as endometrial cell cancer (21), overexpression of gC1qR has been associated with poor prognosis. In addition, gC1qR is being considered as a potential molecular target for delivery of cytotoxic agents (22, 23) in breast cancer.

gC1qR, is a multicompartmental cellular protein (24), with expression in mitochondria, the cytosol, and at the cell surface. In addition, gC1qR is cleaved from cell membrane for release into the extracellular milieu by enzymes such as the membrane-type metalloproteinase MT1MMP (25, 26). gC1qR shedding by cancer cells has been hypothesized to form a biochemical shield to protect malignant cells from complement mediated attack and produce inflammatory mediators to promote and enhance cancer metastasis (27). Interestingly, gC1qR has been described to exert both pro-proliferative and antiproliferative properties in cancer (24). Many cancer cells have been reported to express gC1qR with varying biological effects (11, 13, 28).

We recently described the overexpression of gC1qR in all mesothelioma subtypes, including epitheloid, sarcomatoid, and biphasic phenotypes (29). This finding suggests that gC1qR may represent a novel therapeutic target against mesothelioma. The present study tested this hypothesis in *in vitro* and *in vivo* experiments using a biphasic cultured human mesothelioma cell line, MSTO-211H (MSTO). The data demonstrate that targeting gC1qR with monoclonal antibody 60.11 reduces cell proliferation *in vitro* and tumor growth *in vivo*, associated with increased apoptosis and decreased angiogenesis, and provide proof of concept for further exploration of gC1qR directed therapy in mesothelioma.

## Methods

### Materials

The following materials were purchased from the sources indicated: MSTO-211H biphasic mesothelioma cell line (MSTO; ATCC, Manassas, VA); RPMI 1640, 100× penicillin/streptomycin, and 0.05% trypsin-EDTA (GIBCO-Invitrogen, Grand Island, NY); heat-inactivated fetal bovine serum (FBS; Hyclone, Logan, UT); coating buffer (CB) comprised of 35 mM sodium bicarbonate and 15 mM sodium carbonate, aqueous; tris-buffered saline (TBS) comprised of 20 mM tris-HCl, 150 mM sodium chloride, and 0.05% tween, aqueous; ChromPure human IgG Fc fragments (Jackson ImmunoResearch, West Grove, PA); para-nitrophenyl phosphate (pNPP; Pierce, Rockford, IL); and Dulbecco's PBS (Mediatech Inc., Manassas, VA).

### Monoclonal Antibodies (mAbs)

mAbs to gC1qR were generated as described (30). The 60.11 therapeutic antibody is directed against the C1q binding domain of gC1qR, amino acids 76–93. mAb 74.5.2 recognizes amino acids 204–218, which constitutes the binding site for high molecular weight kininogen. AlexaFluor 488-conjugated secondary antibodies (Invitrogen, Carlsbad, CA) and non-immune mouse IgG MOPC 21 (Sigma-Aldrich) were purchased.

### Cell Culture

MSTO cells were cultured in RPMI supplemented with 10% fetal bovine serum, 100 U/mL penicillin, and 100 μg/mL streptomycin, in T175 culture flasks. Cultures were maintained at 37°C, 100% humidity, 5% CO_2_, and subcultured when cells reached ~90% confluence.

### Recombinant gC1qR

The strategy for the construction of a plasmid containing the full-length (mature form or wild type, WT) and purification of the glutathione-*S*-transferase (GST)–gC1qR fusion products has been described in detail (14). The GST–gC1qR fusion product is cleaved by thrombin (3.2 μg/ml) and the GST-free gC1qR protein is purified on fast protein liquid chromatography (FPLC, Pharmacia) using a Mono-Q ion exchange column. The single peak containing the gC1qR is pooled, concentrated to 1–2 mg/ml, and stored at −80°C in the presence of 50 nM PPACK (d-phenylalanyl-l-prolyl-l-arginine chloromethyl ketone), a specific thrombin inhibitor (Sigma Aldrich).

### Detection of Soluble gC1qR

A qualitative direct ELISA was used to evaluate the presence of soluble gC1qR in MSTO culture medium. MSTO were seeded at 50,000 cells per well and grown for 48 h. Culture supernatants were harvested and centrifuged to remove cellular debris, diluted 1:1 with CB, and incubated in a high-binding microtiter well-plate for 1 h at ambient temperature. Wells were blocked with 1% heat-inactivated bovine serum albumin (BSA) in TBS, 10 min. Immobilized gC1qR was detected with biotinylated immunoaffinity purified polyclonal antibody to gC1qR peptide (144–155) conjugated to alkaline phosphatase, and pNPP substrate. Absorbance was measured at 405 nm.

Soluble gC1qR in pleural fluid from patients with advanced malignant pleural mesothelioma was quantified using a commercial, quantitative human gC1qR ELISA kit (Hycult, Netherlands) (26). Deidentified patient samples (*n* = 22) were evaluated according to manufacturer instruction and in compliance with Memorial Sloan Kettering IRB approved protocols (#16-1547).

### Immunofluorescence Microscopy

MSTO cells were seeded in 24-well-tissue culture-treated plates (50,000 cells per well) and grown to near-confluence. Cells were fixed (10 min) in 1% paraformaldehyde. Wells were blocked with 1% BSA and 1 μg/ml Fc fragments (30 min). gC1qR expression was examined by incubation (30 min) with anti-gC1qR mAb 60.11 (5 μg per well) followed by 30 min incubation with AlexaFluor 488-conjugated goat anti-mouse secondary antibody (5 μg per well). Staining with non-immune rabbit IgG (NIRG) and AlexaFluor 488-conjugated goat anti-rabbit secondary antibody served as a negative control. DAPI (0.2 μg per well) was used as a nuclear counterstain. Images were obtained using an Evos FL Imaging System at 10× magnification and normalized for background brightness. Additional brightness and contrast adjustments were applied uniformly to each image via Adobe Photoshop CS6.

### Flow Cytometry

MSTO cells were detached from culture plates by incubation (30 min, room temperature) with 0.05% trypsin−0.01% EDTA in 0.01 M TBS, centrifuged (800 g, 5 min) and suspended using 10 mM EDTA in PBS, pH 7.4. Cellular Fc receptors were blocked with 5 μg Fc fragments per 500,000 cells. gC1qR expression was detected with mouse anti-gC1qR mAb 74.5.2, and MOPC 21, as negative control. The gC1qR 74.5.2 antibody was preferred for flow cytometry studies as it produced greater staining of cells than the 60.11 antibody. Primary antibodies were visualized with AlexaFluor 488-conjugated goat pAb to mouse IgG. Unstained cells were used an additional negative control. Cells were fixed in 1% paraformaldehyde. All reagents were diluted in DPBS. Fluorescence was determined using a FACSCalibur flow cytometer (BD Biosciences, San Jose, CA).

### MSTO Cell Adhesion Assay

Cells were cultured as described above and imaged at 24-h intervals via compound light microscopy (10× magnification) with a PAXcam 3 microscope camera and Pax-it 11 software (Paxcam, Villa Park, IL). MSTO cells were seeded at 50,000 cells/well, in the presence or absence of 5 μg/ml recombinant gC1qR. To evaluate the effect of immobilized gC1qR on cell adhesion, microtiter wells were incubated overnight (37°C) with 20 μg/ml recombinant gC1qR in CB and rinsed with PBS before exposure to cells.

### MSTO Cell Proliferation Assay

MSTO cells were seeded in 24-well-cell culture plates at 50,000 cells per well and allowed to adhere overnight. Cultures were subsequently treated with 10 μg/ml anti-gC1qR mAb 60.11 or 74.5.2. Untreated cultures were used as controls. Cell proliferation was determined at 24 h intervals. At the desired times, cells were removed from culture wells using 0.05% trypsin-EDTA, stained with trypan blue dye, and counted using a hemocytometer. Trypan blue positive cells were excluded from the count.

### Orthotopic Pleural Mesothelioma Mouse Model

All procedures were performed under approved Institutional Animal Care and Use Committee protocols. Female severe combined immunodeficiency gamma mice (NSG (NOD, scid, gamma), Jackson Laboratories), 6–8 weeks old, were anesthetized using inhaled isoflurane and oxygen. Direct intrapleural injection of 1 × 10^6^ GFP-Firefly Luciferase expressing MSTO-211H cells in 200 μl serum-free media was administered to establish orthotopic malignant pleural mesothelioma tumors via a right thoracic incision, as previously described (31–33). For this proof of concept study, mice were divided into two treatment groups: vehicle (*n* = 10), and 60.11 antibody treatment (*n* = 10) (100 mg/kg, administered twice weekly by intraperitoneal injection beginning on day 8 after tumor implantation). Intraperitoneal injection of antibody was chosen over the intravenous route, as it allowed for the administration of greater fluid volume, which was required to reach the desired therapeutic antibody dose. Clinical assessments of animal distress (weight loss, disruption of locomotor coordination, hunching, lack of grooming, lethargy) were made and recorded daily to assess toxicity.

Tumor burden was measured beginning on day 8 after tumor implantation, and at regular intervals until day 23 using quantitative bioluminescence imaging (BLI). Mice were imaged with the Xenogen IVIS 100 Imaging System, 20 min following injection of a single intraperitoneal dose of 150 mg/kg D-Luciferin. Images were acquired for 5–30 s depending on signal strength. BLI data were analyzed using Living Image 2.60 software and BLI signal reported as total flux (Photons/s). Due to high tumor burden observed in the control group, the experiment was terminated at day 23. At time of sacrifice, blood was collected for cell counts and measurement of serum 60.11 antibody levels using a direct ELISA assay with immobilized recombinant gC1qR. Tumors were removed, fixed and processed for histologic (hematoxylin and eosin staining) and immunohistochemical evaluation.

### Immunohistochemical Analysis

Tissue processing and immunohistochemical analysis was performed by the Molecular Cytology Core Facility of Memorial Sloan Kettering Cancer Center as previously described (31, 32). In brief, tissues were fixed in 4% formaldehyde and processed by paraffin embedding using a tissue processor (Leica ASP6025). Five micrometer sections were obtained and applied to superfrost plus slides. Immunohistochemical detection of Ki67, Cleaved Caspase 3, TUNEL, and CD31 was performed using a Discovery XT processor (Ventana Medical Systems). Slides were counterstained with hematoxylin and cover-slipped with Permount (Fisher Scientific).

### Ki-67

The Discovery XT autostainer was programmed to incubate slides with primary rabbit polyclonal Ki-67 antibody (Abcam, catalog # ab16667) at 1 μg/ml for 4 h, followed by incubation with secondary antibody (biotinylated goat anti-rabbit IgG; Vector labs) at a concentration of 5.75 μg/ml for 30 min. Blocker D, Streptavidin-HRP, and DAB detection kit (Ventana Medical Systems) were used according to manufacturer instructions.

### Cleaved Caspase 3

A rabbit polyclonal Cleaved Caspase 3 antibody (Cell Signaling, catalog # 9661) was used at 0.1 μg/ml concentration. Slides were incubated in the Discovery XT autostainer for 3 h. Incubation with secondary antibody (biotinylated goat anti-rabbit IgG; Vector labs) at a concentration of 5.75 μg/ml occurred for 20 min. Blocker D, Streptavidin-HRP, and DAB detection kit (Ventana Medical Systems) were used according to manufacturer instructions.

### TUNEL

Terminal deoxynucleotidyl dUTP nick end labeling (TUNEL) analysis was done as follows. Slides were manually deparaffinized in xylene, rehydrated in a series of alcohol dilutions (100, 95, and 70%) and tap water, and placed into the autostainer, where tissue sections were treated with Proteinase K (20 μg/ml in PBS) for 8 min, and incubated with endogenous biotin blocking kit (Roche) for 12 min, followed by incubation with labeling mix: TdT (Roche, 1,000 U/ml) and biotin-dUTP (Roche, 4.5 nmol/ml) for 2 h. Detection was performed with Streptavidin-HRP and DAB detection kit (Ventana Medical Systems) according to the manufacturer's instruction.

### CD31

Primary antibody, a rat anti-mouse CD31 antibody (Dianova, catalog # DIA-310) was used at 2 μg/ml. Slides were incubated in the autostainer for 6 h, followed by exposure to biotinylated rabbit anti-rat IgG (Vector, 1:200 dilution) for 60 min. Blocker D, Streptavidin-HRP, and DAB detection kit (Ventana Medical Systems) were used according to the manufacturer's instructions.

### Quantitative Analysis of Immunohistochemical Staining

Quantitative analysis of immunohistochemical staining was performed of images generated by a slide scanner (Panoramic Flash 250, 3DHistech, Hungary) using Image J software.

### Statistical Analysis

Data were analyzed with Student's *t*-test as applicable, and *p* < 0.05 were considered statistically significant. All statistical analysis, calculations, and graphing were performed in Excel (Microsoft, Redmond, WA).

## Results

### *In vitro* Studies

**Figure 1:**
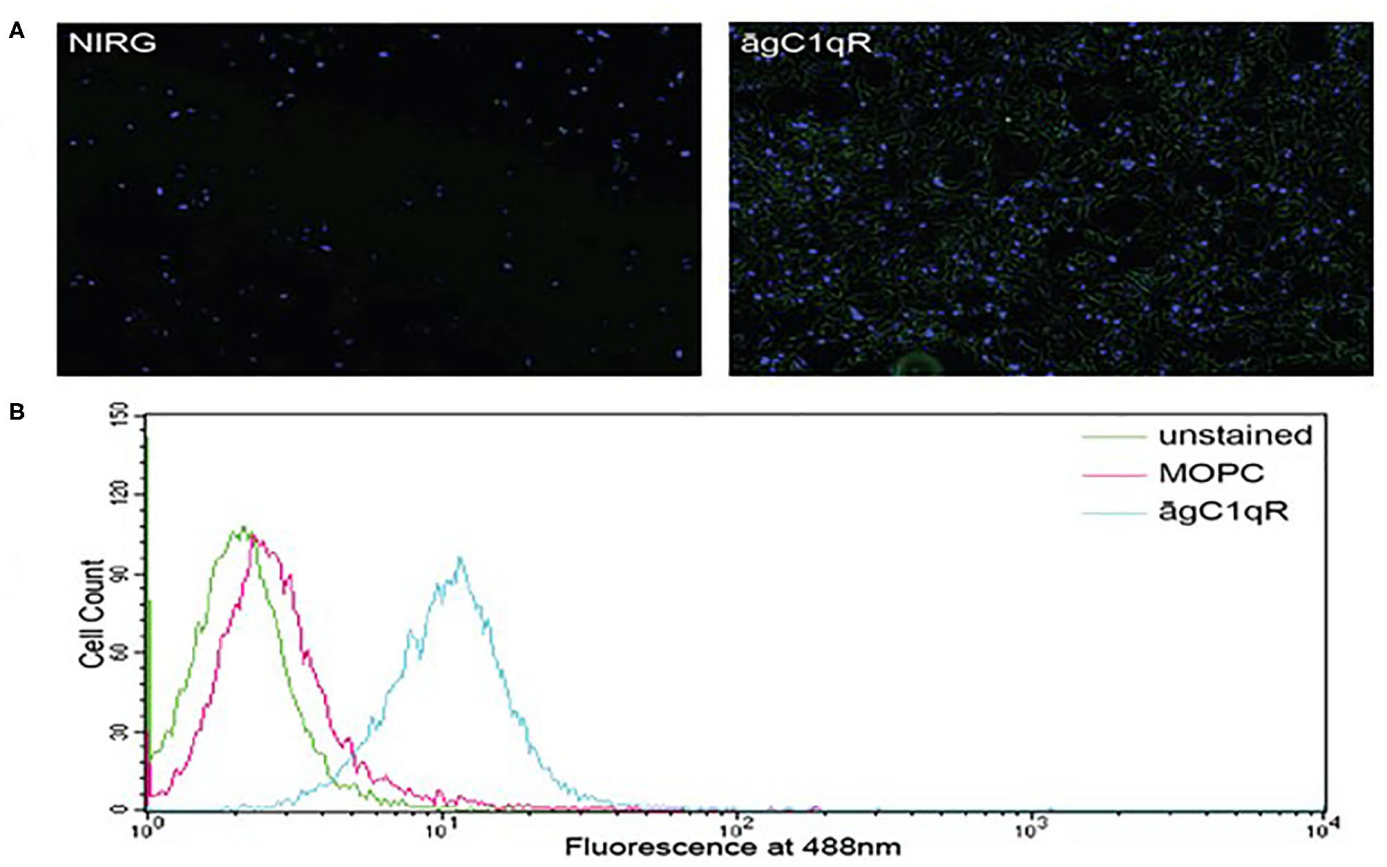
Mesothelioma MSTO-211H cells expresses gC1qR. **(A)** Immunofluorescence photomicrographs of stained MSTO-211H cells in culture. Results of a typical experiment. Non-permeabilized cell monolayers were stained with non-immune rabbit IgG (NIRG) or 60.11 anti gC1qR antibody (āgC1qR) and appropriate secondary antibodies conjugated to AlexaFluor 488 (green), as well as DAPI nuclear stain (blue). Cells were imaged with a fluorescence microscope at 10× magnification. The presence of gC1qR is shown by green staining in the right image. **(B)** Flow cytometric analysis of MSTO-211H cell surface gC1qR expression. Non-permeabilized MSTO-211H cells in suspension were stained with non-immune mouse IgG (MOPC) or anti gC1qR antibody (āgC1qR) and appropriate secondary antibodies conjugated to AlexaFluor 488. Stained and unstained cells were analyzed. Expression of cell surface gC1qR in a typical experiment is shown by an increase in fluorescence of cells treated with anti gC1qR antibody (blue curve).

**Figure 2:**
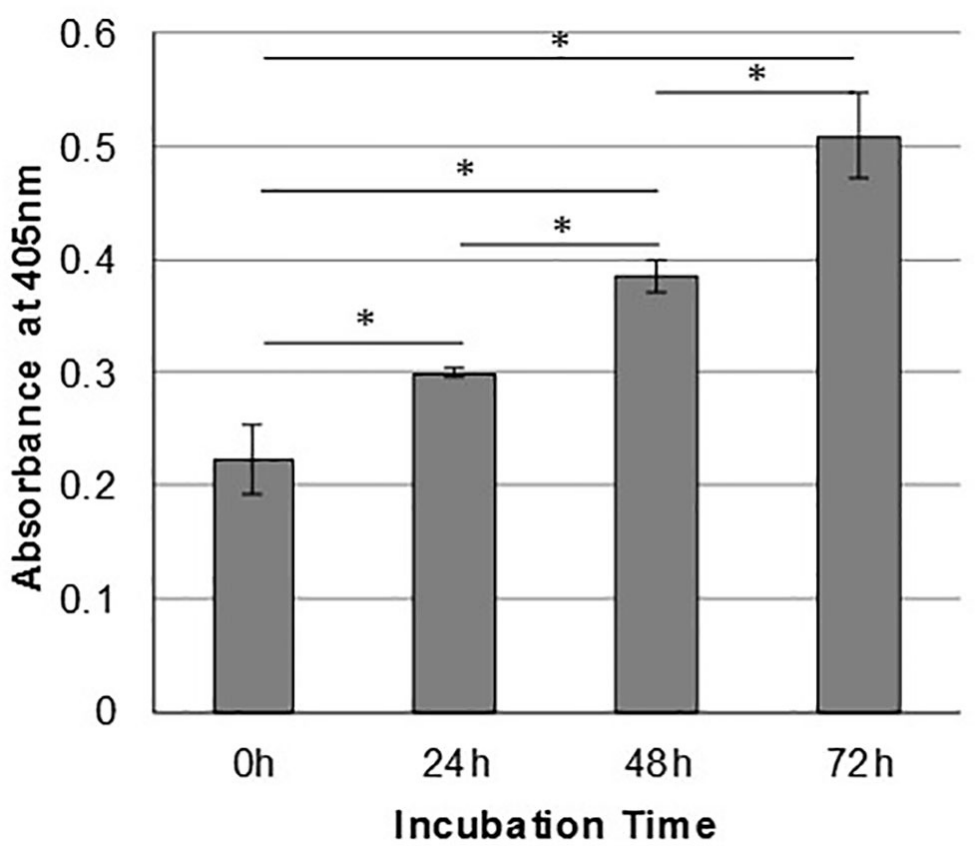
Mesothelioma cells shed gC1qR into the extracellular milieu. MSTO-211H cells were cultured in complete medium, and supernatants were sampled at 24-h intervals. Relative concentrations of gC1qR in the supernatants at each time point were determined via a direct ELISA. Error bars represent mean substrate absorbance ± standard deviation. *n* = 2 separate experiments performed in duplicate. *Significantly different results at *p* < 0.05.

**Figure 3:**
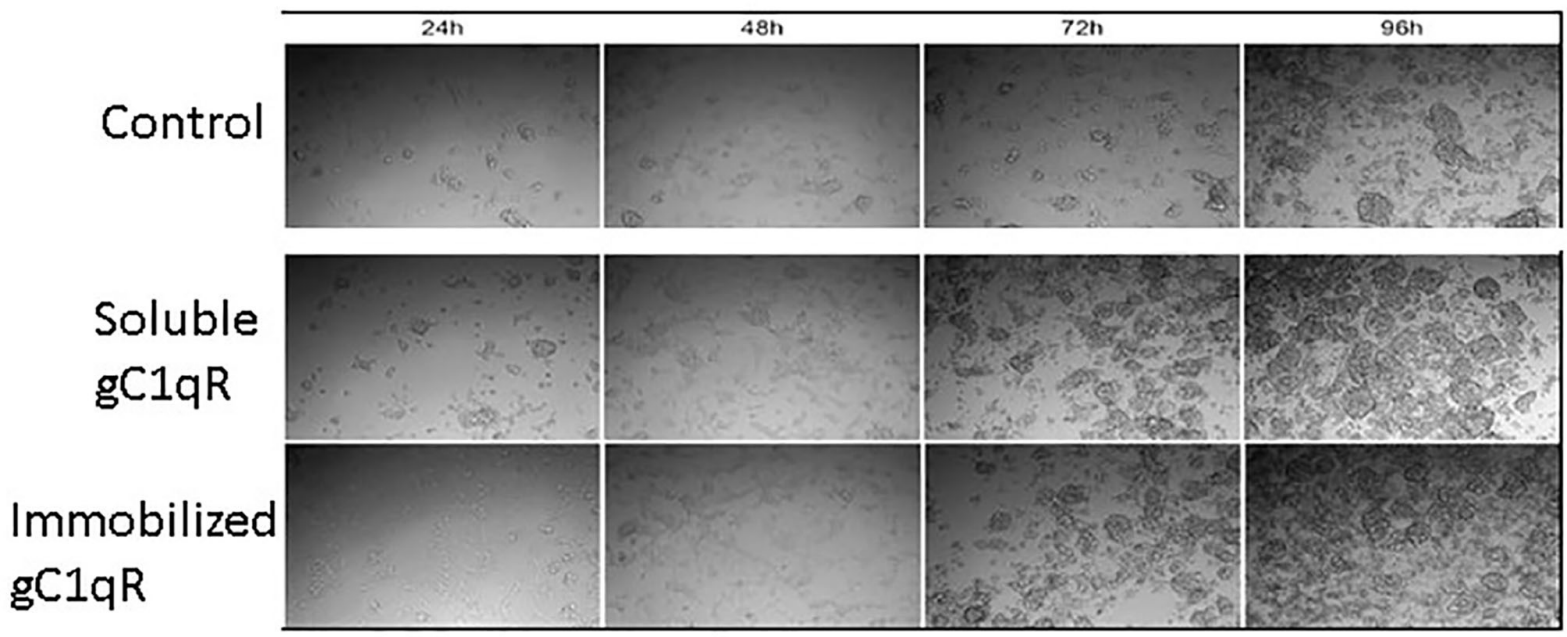
Extracellular gC1qR enhances MSTO-211H cell growth in culture. Tissue culture wells were coated with gC1qR or coating buffer (control) and seeded with MSTO-211H cells. Cells were supplemented with recombinant gC1qR (5 μg/ml) as noted. Cell cultures were imaged at 24-h intervals via compound light microscopy (10× magnification). Representative images are shown. Increased cell growth is evident at 72 and 96 h in the presence of soluble or immobilized extracellular gC1qR.

**Figure 4:**
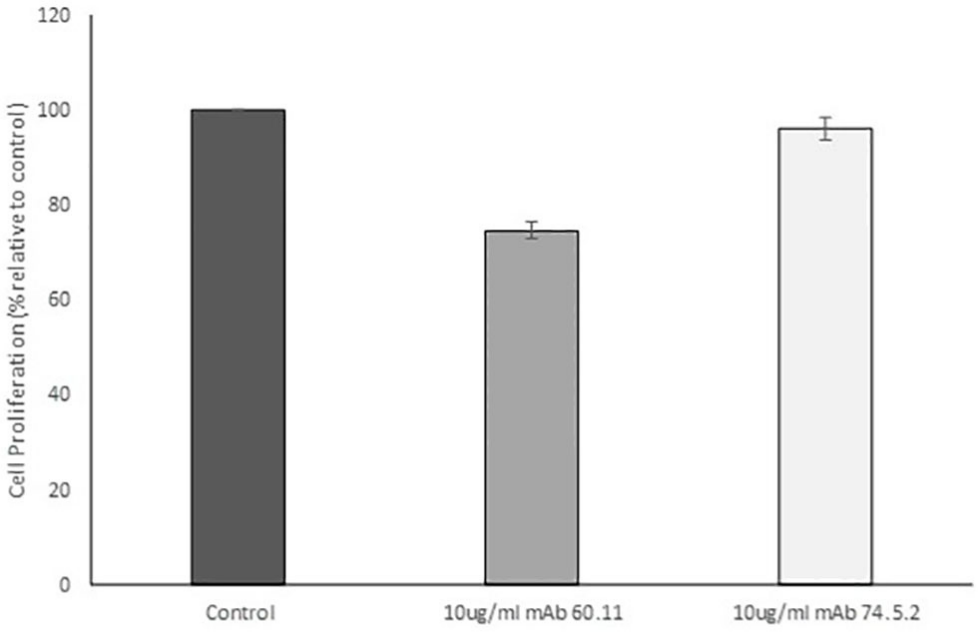
Antibodies directed against the C1q binding site of gC1qR decrease mesothelioma cell proliferation. MSTO-211H cells was treated with 10 μg/mL monoclonal anti gC1qR antibody mAb 60.11 directed against the C1q binding region (aa 76–93) or mAb 74.5.2 directed against the high molecular weight kininogen binding domain (aa 204–218). Cell proliferation was determined via hemocytometer cell counts of viable cells after 96 h incubation. Error bars represent mean cell population ± standard deviation. *n* = 2 separate experiments performed in duplicate. *Significant difference from control at *p* = 1.33 × 10^−5^.

In order to assess the effect of anti-gC1qR therapy on MSTO cell proliferation, *in vitro* studies first evaluated the expression of the target antigen by MSTO cells. Expression of gC1qR by MSTO cells was demonstrated by immunofluorescence microscopy (Figure 1A) and flow cytometry (Figure 1B). In addition, soluble gC1qR was detected in culture supernatants of MSTO cells. Progressively increasing amounts of gC1qR were shed into the cell culture medium over a 72 h time course (Figure 2). Soluble gC1qR was found also i*n vivo*, in 10 of 22 pleural fluids from patients with malignant pleural mesothelioma, with a mean concentration of 1.11 ± 0.57 ng/ml (*n* = 10). Interestingly, soluble gC1qR and immobilized gC1qR, enhanced MSTO cell adhesion in culture (Figure 3). Cell proliferation was reduced 25.3 ± 1.8% (*n* = 4) by targeting gC1qR with mAb (60.11) (Figure 4). This inhibition was specific for mAb 60.11, directed against amino terminal amino acids 76–93, representing the C1q binding domain (28). As illustrated in Figure 4, gC1qR mAb 74.5.2, directed against aa 204–218, had a negligible effect (2.9 ± 2.2% inhibition).

### *In vivo* Studies

An orthotopic mouse model of malignant pleural mesothelioma was used to evaluate gC1qR blockade with mAb 60.11 on MSTO cell proliferation. The data are summarized in Table 1. Animals treated with the 60.11 antibody showed an approximately 50% reduction in tumor development compared to vehicle control. Serum 60.11 antibody concentrations measured on day of sacrifice ranged from 30 to 50 μg/ml. 60.11 therapy was not associated with clinical changes such as weight loss (Table 2), disruption of locomotor coordination, hunching, and lack of grooming or lethargy. Comparison of peripheral blood cell counts showed no change in RBC and platelet counts between treatment groups, but a modest decrease in WBC counts, predominantly associated with a decrease in neutrophils in 60.11 treated mice, was observed (Table 2).

**Table 1:** Targeted gC1qR (60.11) treatment reduces MSTO-211H tumor cell growth in an orthotopic murine xenotransplant model.

	**Treatment Group**		
	**Tumor Burden (BLI, Total Flux, × 10^7^)**		
	**Vehicle Control**	**60.11 Treatment**	***p***
Baseline	2.1 ± 1.0	2.4 ± 1.4	0.635
Week 1	18.8 ± 6.1	9.6 ± 3.6	0.0006
Week 2	61.1 ± 25.6	34.6 ± 19.6	0.018

*BLI, Bioluminescence Imaging*.

**Table 2:** Effect of 60.11 therapy on mouse weight and blood cell counts.

	**Treatment Groups**	
	**Vehicle**	**60.11 treatment**
**Weight (g)**	23.0 ± 1.16	23.5 ± 1.08
**Cell Count**		
RBC (10^6^/μl)	9.01 ± 0.70	8.58 ± 0.63
Hgb (g/dl)	15.01 ± 1.15	14.23 ± 1.01
HCT (%)	43.54 ± 3.14	41.91 ± 2.50
MCV (fl)	48.33 ± 0.61	48.92 ± 1.13
Platelets (10^3^/μl)	1579 ± 121	1544 ± 172
WBC (10^3^/μl)	5.02 ± 1.05	3.65 ± 1.02^*^
Neutrophils (10^3^/μl)	4.57 ± 0.92	3.24 ± 0.91^*^
Lymphocytes (10^3^/μl)	0.032 ± 0.019	0.014 ± 0.014^*^
Monocytes (10^3^/μl)	0.292 ± 0.134	0.258 ± 0.10
Eosinophils (10^3^/μl)	0.125 ± 0.067	0.137 ± 0.081
Basophils (10^3^/μl)	0.06 ± 0.013	0.02 ± 0.004

* *P < 0.05*.

**Figure 5:**
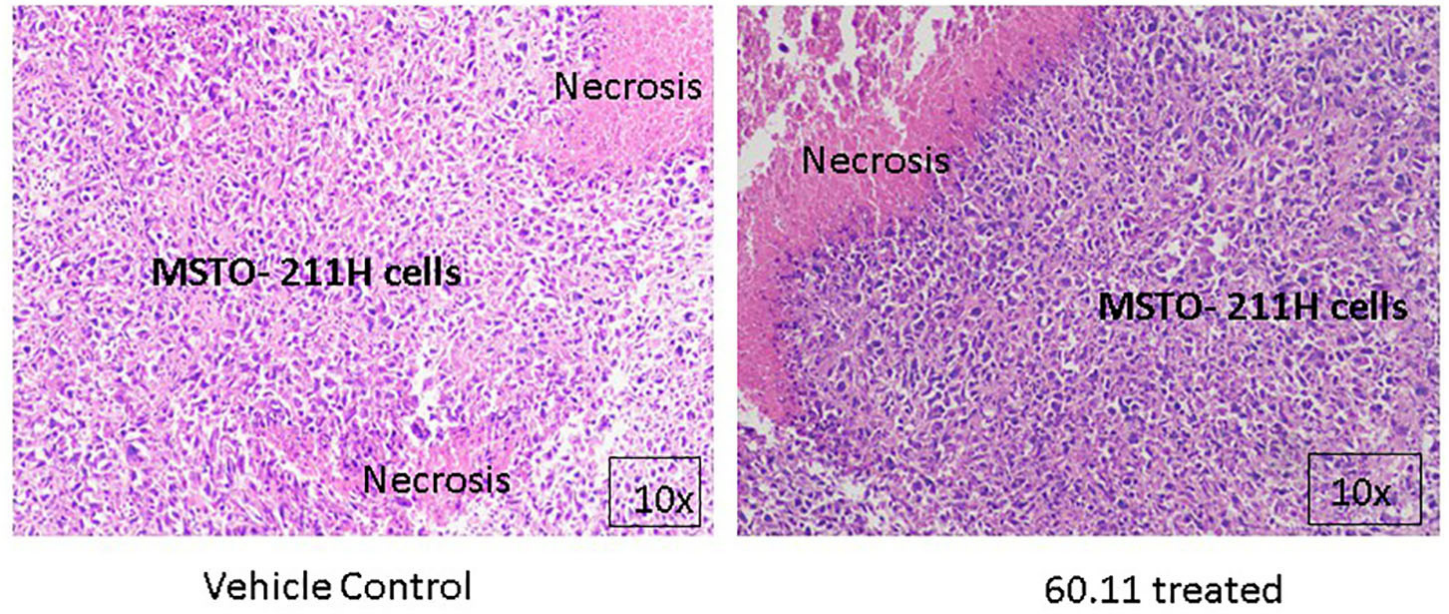
MSTO-211H tumors resected from control and 60.11 treated mice are histologically similar. Representative histologic (10×) images of tumors stained with hematoxylin and eosin show tightly packed MSTO-211H cells and areas of necrosis. No histologic differences in tumor morphology were apparent between control and treatment groups.

**Figure 6:**
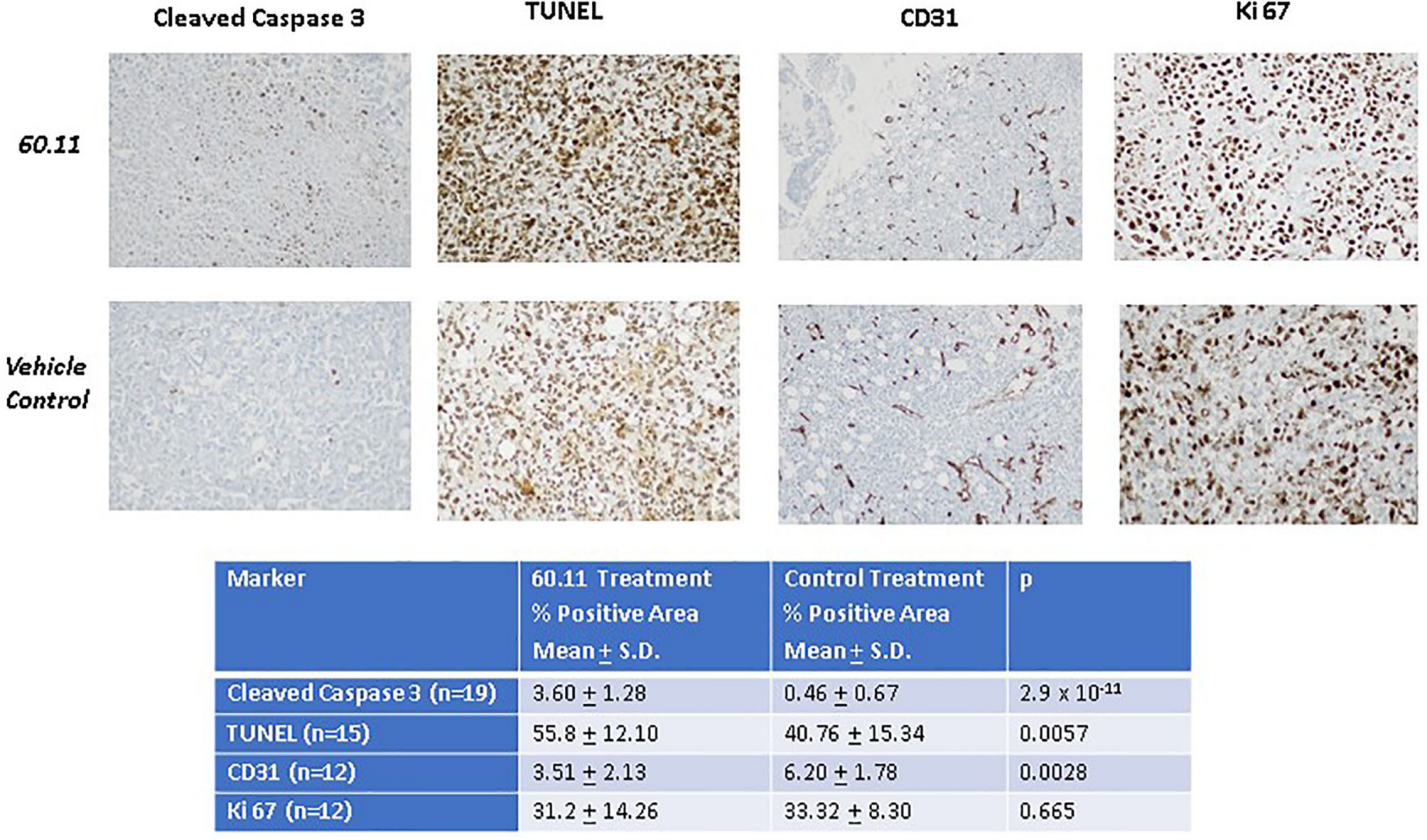
Therapy with 60.11 increases apoptosis and reduces neovascularization in mesothelioma. Representative histologic images (20 × original magnification) of tumors obtained from control and 60.11 treated mice stained with cleaved caspase 3, TUNEL, CD 31, and Ki 76 are shown. Positive immunohistochemical reactivity is indicated by brown stain. Quantitative analysis of staining intensity is shown in the inset. *N*, number of microscopic fields selected for analysis.

Figure 5 shows representative images of tumors resected from control and 60.11 treated mice. Although tumors from 60.11 treated mice were macroscopically smaller than those excised from control mice, no histologic differences were appreciated by H&E staining. Tumors consisted of densely packed MSTO cells with significant areas of necrosis. Immunohistochemical analysis (Figure 6) revealed an increase in early and late apoptosis markers, cleaved caspase 3, and TUNEL, respectively, in tumors from 60.11 treated mice compared to controls. No difference in the tumor cell proliferation index (Ki67) was noted between treatment groups. Interestingly, 60.11 therapy was associated with decreased tumor CD31 staining, suggesting decreased angiogenesis. In addition, the CD31 positive vessels in tumors of 60.11 treated animals appeared generally small and poorly developed.

## Discussion

The data provide proof of concept that targeting the complement receptor, gC1qR, at the C1q binding site, may provide a potential novel therapeutic strategy in mesothelioma. The present study was developed based on our recent observation that gC1qR is overexpressed in malignant pleural mesothelioma (29), and reports from breast and lung cancer models, indicating decreased tumor cell proliferation (34–36) using gC1qR-targeted strategies. *In vitro* studies with the biphasic mesothelioma cell line MSTO demonstrate the presence of targetable gC1qR on the cell surface and in the extracellular milieu. Interestingly, extracellular gC1qR enhanced MSTO cell adhesion and proliferation *in vitro*, and may have similar direct effects *in vivo*. Additional postulated roles for extracellular gC1qR include shielding of the tumor from classical complement mediated attack, and activation of the kallikrein system with bradykinin generation and vascular leakage that may contribute to tumor metastasis (27).

The ability of tumor cells to adhere to tissue surfaces is a key element in metastasis formation (37). Thus, the enhanced ability of MSTO cells to adhere to immobilized gC1qR supports the hypothesis that gC1qR in the tumor microenvironment may contribute to tumor progression via autocrine or paracrine effects. This observation requires further study, since levels of soluble gC1qR, previously detected in blood and body fluids (26), as well as those noted in pleural effusions from patients with MPM, are significantly lower than those found to enhance cell proliferation *in vitro*. Concentrations in the tumor microenvironment, however, are likely different, and greater in the microenvironment immediately adjacent to the tumor.

Targeting gC1qR, at the C1q binding site with mAb 60.11, reduced MSTO cell proliferation *in vitro*, and to a greater extent *in vivo*, using a clinically relevant orthotopic pleural mesothelioma mouse model. This model resembles pleural mesothelioma in humans with associated extensive lymphangiogenesis, regional invasion, and shortened survival (31). Data from immunohistochemical studies comparing tumors from 60.11 treated and control mice reveal that inhibition of *in vivo* mesothelioma tumor growth is associated with both increased apoptosis and decreased angiogenesis.

One of the major ligands of gC1qR is C1q (14). The role of C1q in tumorigenesis is multifactorial. C1q is thought to promote tumor growth in part through its support of neovascularization (12). Indeed, in the present study, immunohistochemical analysis of tumors treated with 60.11 therapy demonstrate a decreased and abnormal microvasculature. Since mAb 60.11 is directed specifically against the C1q binding site of gC1qR, these findings support the hypothesis that gC1qR—C1q interactions in the tumor cell microenvironment contribute to mesothelioma tumor growth. Interestingly, recent immunohistochemical studies report the presence of C1q in mesothelioma (38). The observed anti-angiogenic effect of 60.11 therapy likely contributes to the overall *in vivo* effect of 60.11 therapy. This observation illustrates the complexity of tumor growth *in vivo*, and demonstrates that cell proliferation *in vitro* is not directly comparable to tumor growth *in vivo*.

Therapy with 60.11 was not associated with clinically discernable toxicity. Animal weights were similar between treatment and control groups. No difference in animal appearance, habitus or behavior was noted. However, a statistically significant decrease in total WBC was observed in the treatment group, which was attributed predominantly to a decrease in neutrophil count. Lymphocyte counts in the NSG mice are extremely low and differences between groups are therefore difficult to interpret. Given the observed significant anti-mesothelioma effect of 60.11 therapy, further exploration of both its therapeutic potential and toxicity profile are indicated.

The paucity of effective therapies for mesothelioma, including the limited success of mesothelin targeted therapy, immune checkpoint blockade, anti-angiogenesis therapies, and neoantigen based vaccines (39), necessitate the development of additional therapeutic strategies. This exploratory study provides the first *in vivo* proof of concept that targeting gC1qR at the C1q binding site can significantly reduce mesothelioma tumor burden by increasing tumor cell apoptosis and decreasing tumor angiogenesis. The study is limited by use of a single cell line and a single anti gC1qR targeting strategy. However, current results support further exploration of gC1qR as a potential new therapeutic target. Investigation of additional gC1qR targeting antibodies may further maximize treatment efficacy. For example, the 74.5.2 antibody, which has no effect on cell proliferation, is a potent inhibitor of vascular permeability, blocking the binding of high molecular weight kininogen to vascular endothelial cells (40). In addition, combining gC1qR targeted therapy with chemotherapy or other targeted therapies should be evaluated in further preclinical studies.

## Data Availability Statement

The raw data supporting the conclusions of this article will be made available by the authors, without undue reservation.

## Ethics Statement

The studies involving human participants were reviewed and approved by Institutional Review Board/Privacy Board (FWA00004998). Written informed consent for participation was not required for this study in accordance with the national legislation and the institutional requirements. The animal study was reviewed and approved by Institutional Animal Care and Use Committee of Memorial Sloan Kettering Cancer Center (protocol no. 04-03-009).

## Author Contributions

EP, BG, and PA designed the study, supervised experiments, analyzed data, and wrote the manuscript. ES, QC, and YX conducted the *in vivo* studies. KM-T, NF, and AB performed immunohistochemical analyses. KS and EK performed *in vitro* studies. All authors participated in data review and review of the manuscript.

## Conflict of Interest

EP and BG receive royalty income from the commercialization of gC1qR antibodies, including 60.11, and hold a licensed patent for the use of gC1qR antibodies. PA has received research funding from ATARA Biotherapeutics and OSE Immunotherapies. The remaining authors declare that the research was conducted in the absence of any commercial or financial relationships that could be construed as a potential conflict of interest.

## References

[1] Mossman BT Churg A. Mechanisms in the pathogenesis of asbestosis and silicosis. Am J Respir Crit Care Med. (1998) 157:1666–80. 10.1164/ajrccm.157.5.9707141 9603153

[2] Adusumilli PS. Translational immunotherapeutics: chemoimmunotherapy for malignant pleural mesothelioma. Cancer. (2014) 120:3268–71. 10.1002/cncr.28883 24989696 PMC4448967

[3] Krug LM Pass HI Rusch VW Kindler HL Sugarbaker DJ Rosenzweig KE. Multicenter phase II trial of neoadjuvant pemetrexed plus cisplatin followed by extrapleural pneumonectomy and radiation for malignant pleural mesothelioma. J Clin Oncol. (2009) 27:3007–13. 10.1200/JCO.2008.20.3943 19364962 PMC3646305

[4] Nelson DB Rice DC Niu J Atay S Vaporciyan AA Antonoff M. Long-term survival outcomes of cancer-directed surgery for malignant pleural mesothelioma: Propensity score matching analysis. J Clin Oncol. (2017) 35:3354–62. 10.1200/JCO.2017.73.8401 28817374

[5] Vogelzang NJ Rusthoven JJ Symanowski J Denham C Kaukel E Ruffie P. Phase III study of pemetrexed in combination with cisplatin versus cisplatin alone in patients with malignant pleural mesothelioma. J Clin Oncol. (2003) 21:2636–44. 10.1200/JCO.2003.11.136 12860938

[6] Odgerel C-O Takahashi K Sorahan T Driscoll T Fitzmaurice C Yoko-O M. Estimation of the global burden of mesothelioma deaths from incomplete national mortality data. Occup Environ Med. (2017) 74:851–8. 10.1136/oemed-2017-104298 28866609 PMC5740549

[7] Lioy PJ Weisel CP Millette JR Eisenreich S Vallero D Offenberg J. Characterization of the dust/smoke aerosol that settled east of the World Trade Center (WTC) in lower Manhattan after the collapse of the WTC 11 September 2001. Environ Health Perspect. (2002) 110:703–14. 10.1289/ehp.02110703 12117648 PMC1240917

[8] Scott AM Allison JP Wolchok JD. Monoclonal antibodies in cancer therapy. Cancer Immun. (2012) 12:14. 10.1038/nrc3236 22896759 PMC3380347

[9] Drewe E Powell RJ. Clinically useful monoclonal antibodies in treatment. J Clin Pathol. (2002) 55:81–5. 10.1136/jcp.55.2.81 11864998 PMC1769580

[10] Kourtzelis I Rafail S. The dual role of complement in cancer and its implication in anti-tumor therapy. Ann Transl Med. (2016) 4:265. 10.21037/atm.2016.06.26 27563652 PMC4971376

[11] Bulla R Tripodo C Rami D Ling GS Agostinis C Guarnotta C. C1q acts in the tumor microenvironment as a cancer-promoting factor independently of complement activation. Nat Commun. (2016) 7:10346 10.1038/ncomms10346 26831747 PMC4740357

[12] Bossi F Tripodo C Rizzi L Bulla R Agostinis C Guarnotta C. C1q as a unique player in angiogenesis with therapeutic implication in wound healing. Proc Natl Acad Sci U S A. (2014) 111:4209–14. 10.1073/pnas.1311968111 24591625 PMC3964125

[13] Ghebrehiwet B Hosszu KK Valentino A Peerschke EIB. The C1q family of proteins: insights into the emerging non-traditional functions. Front Immunol. (2012) 3:52. 10.3389/fimmu.2012.00052 22536204 PMC3334295

[14] Ghebrehiwet B Lim BL Peerschke EIB Willis AC Reid KBM. Isolation, cDNA cloning, and overexpression of a 33-kDA cell surface glycoprotein that binds to the globular heads of C1q. J Exp Med. (1994) 179:1809–21. 10.1084/jem.179.6.1809 8195709 PMC2191527

[15] Rubinstein DB Stortchevoi A Boosalis M Ashfaq R Ghebrehiwet B Peerschke EI. Receptor for the globular heads of C1q (gC1q-R, p33, hyaluronan binding protein) is preferentially expressed by adenocarcinoma cells. Intern J Cancer. (2004) 110:741–50. 10.1002/ijc.20105 15146564

[16] Dembitzer FR Kinoshita Y Burstein D Phelps RG Beasley MB Garcia R. gC1qR expression in normal and pathologic human tissues: differential expression in tissues of epithelial and mesenchymal origin. J Histochem Cytochem. (2012) 60:467–74. 10.1369/0022155412440882 22638269 PMC3393077

[17] Chen YB Jiang CT Zhang GQ Wang JS Pang D. Increased expression of hyaluronic acid binding protein 1 is correlated with poor prognosis in patients with breast cancer. J Surg Oncol. (2009) 100:382–6. 10.1002/jso.21329 19565630

[18] Jiang Y Wu H Liu J Chen Y Xie J Zhao Y. Increased breast cancer risk with HABP1/p32/gC1qR genetic polymorphism rs2285747 and its upregulation in northern Chinese women. Oncotarget. (2017) 8:13932–41. 10.18632/oncotarget.14737 28108744 PMC5355151

[19] Amamoto R Yagi M Song Y Oda Y Tsuneyoshi M Naito S. Mitochondrial p32/C1QBP is highly expressed in prostate cancer and is associated with shorter prostate-specific antigen relapse time after radical prostatectomy. Cancer Sci. (2011) 102:639–47. 10.1111/j.1349-7006.2010.01828.x 21205079

[20] Yu G Wang J. Significance of hyaluronan binding protein (HABP1/P32/gC1qR) expression in advanced serous ovarian cancer patients. Exp Mol Pathol. (2013) 94:210–5. 10.1016/j.yexmp.2012.06.007 22771308

[21] Zhao J Liu T Yu G Wang J. Overexpression of HABP1 correlated with clinicopathological characteristics and unfavorable prognosis in endometrial cancer. Tumour Biol. (2015) 36:1299–306. 10.1007/s13277-014-2761-8 25355598

[22] Fogal V Zhang L Krajewski S Ruoslathi E. Mitochondria/cell surface protein p32/gC1qR as a molecular target in tumor cells and tumor stroma. Cancer Res. (2008) 7210–8. 10.1158/0008-5472.CAN-07-6752 18757437 PMC2562323

[23] Paasonen L Sharma S Braun GB Katamraju VR Chung TDY She Z. New p32/gC1qR ligands for targeted drug delivery. Chembiochem. (2016) 17:570–5. 10.1002/cbic.201500564 26895508 PMC5433940

[24] Saha P Datta K. Multifunction, multicompartmental hyaluronan-binding protein 1 (HABP1/p32/gC1qR: Implication in cancer progression and metastasis. Oncotarget. (2018) 9:10784–807. 10.18632/oncotarget.24082 29535843 PMC5828189

[25] Rozanov DV Ghebrehiwet B Postnova TI Eichinger A Deryugina EI Strongin AY. The hemopexin-like C-terminal domain of membrane type 1 matrix metalloproteinase regulates proteolysis of a multifunctional protein, gC1qR. J Biol Chem. (2002) 277:9318–25. 10.1074/jbc.M110711200 11773076

[26] Peerschke EIB Brandwijk RJMGE Dembitzer FR Kinoshito Y Ghebrehiwet B. Soluble gC1qR in blood and body fluids: examination in a pancreatic cancer patient cohot. Int J Cancer Res Mol Mech. (2015) 1:10. 10.16966/2381-3318.110 26973884 PMC4786181

[27] Peerschke EIB Ghebrehiwet B. cC1qR/CR and gC1qR/p33: Observations in cancer. Mol Immunol. (2014) 61:100–9. 10.1016/j.molimm.2014.06.011 25044096

[28] Ghebrehiwet B Jesty J Xu S Vinayagasundaram R Vinayagasundaram U Ji Y. Structure-function studies using deletion mutants identify domains of gC1qR/p33 as potential therapeutic targets for vascular permeability and inflammation. Front Immunol. (2011) 2:58. 10.3389/fimmu.2011.00058 22282702 PMC3265123

[29] Li X Eguchi T Aly RG Chintala NK Tan KS Zauderer. Globular C1q receptors (gC1qR/p32/HABP1) is overexpressed in malignant pleural mesothelioma and is associated with increased survival in surgical patients treated with chemotherapy. Front Oncol. (2019) 9:1042. 10.3389/fonc.2019.01042 31681580 PMC6799080

[30] Ghebrehiwet B Lu PD Zhang W Lim BL Eggleton P Leigh LE. Identification of functional domains on gC1Q-R, a cell surface protein that binds to the globular “heads” of C1Q, using monoclonal antibodies and synthetic peptides. Hybridoma. (1996) 15:333–42. 10.1089/hyb.1996.15.333 8913782

[31] Servais EL Suzuki K Colovos C Rodriguez L Sima C Fleisher M. An *in vivo* platform for tumor biomarkder assessment. PLoS One. (2011) 6:e26722. 10.1371/journal.pone.0026722 22046338 PMC3202552

[32] Servais EL Colovos C Rodriguez L Bograd AJ Nitadori J Sima C. Mesothelin overexpression promotes mesothelioma cell invasion and MMP-9 secretion in an orthotopic mouse model and in epithelioid pleural mesothelioma patients. Clin Cancer Res. (2012) 18:2478–89. 10.1158/1078-0432.CCR-11-2614 22371455 PMC3759995

[33] Adusumilli PS Cherkassky L Villena-Vargas J Colovos C Servais E Plotkin J. Regional delivery of mesothelin-targeted CAR T cell therapy generates potent and long-lasting CD4-dependent tumor immunity. Sci Transl Med. (2014) 6:261ra51. 10.1126/scitranslmed.3010162 25378643 PMC4373413

[34] Kandov E Kaur A Kishore U Ji P Williams J Peerschke EIB C1q and C1q receptors (gC1qR and cC1qR) as potential novel targets for therapy against breast cancer. Curr Trends Immunol. (2018)19:59–76. Available online at: https://www.scopus.com/inward/record.uri?eid=2-s2.0-85062166884&partnerID=40&md5=7cbdf37967cb340d8d1f6a75773c4557

[35] Kim KB Yi JS Nguyen N Lee JH Kwon YC Ahn BY. Cell-surface receptor for complement component C1q (gC1qR) is a key regulator for lamellipodia formation and cancer metastasis. J Biol Chem. (2011) 286:23093–101. 10.1074/jbc.M111.233304 21536672 PMC3123076

[36] Kim B-C Hwang H-J An H-T Lee H Park JS Hong J. Antibody neutralization of cell-surface gC1qR/HABP1/SF2-p32 prevents lamellipodia formation and tumorigenesis. Oncotarget. (2016) 7:49972–85. 10.18632/oncotarget.10267 27363031 PMC5226562

[37] Varani J Lovett EJ Elgebaly S Lundy J Ward PA. *In vitro* and *in vivo* adherence of tumor cell variants correlated with tumor formation. Am J Pathol. (1980) 101:345–52. 7435542 PMC1903598

[38] Agostinis C Vidergar R Belmonte B Mangogna A Amadio L Geri P. Complement protein C1q binds to hyaluronic acid in the malignant pleural mesothelioma microenvironment and promotes tumor growth. Front Immunol. (2017) 8:1559. 10.3389/fimmu.2017.01559 29209316 PMC5701913

[39] Mutti L Peikert T Robinson BWS Scherpereel A Tsao AS de Perrot M. Scientific advances and new frontiers in mesothelioma therapeutics. J Thorac Oncol. (2018) 13:1269–83. 10.1016/j.jtho.2018.06.011 29966799 PMC6643278

[40] Fandaros M Ong CL Rubenstein DA Joseph K Kaplan AP Yin W Angioedema and shear stress modulate endothelial permeability through gC1qR. FASEB J. (2019) 33(Suppl 1):542.15.

